# Syngas Fermentation for the Production of Bio-Based Polymers: A Review

**DOI:** 10.3390/polym13223917

**Published:** 2021-11-12

**Authors:** Nirpesh Dhakal, Bishnu Acharya

**Affiliations:** Department of Chemical and Biological Engineering, 57 Drive, University of Saskatchewan, Saskatoon, SK S7N 5A9, Canada; pyt394@mail.usask.ca

**Keywords:** syngas, fermentation, polyhydroxyalkanoates, carbon monoxide dehydrogenase, hydrogenase

## Abstract

Increasing environmental awareness among the general public and legislators has driven this modern era to seek alternatives to fossil-derived products such as fuel and plastics. Addressing environmental issues through bio-based products driven from microbial fermentation of synthetic gas (syngas) could be a future endeavor, as this could result in both fuel and plastic in the form of bioethanol and polyhydroxyalkanoates (PHA). Abundant availability in the form of cellulosic, lignocellulosic, and other organic and inorganic wastes presents syngas catalysis as an interesting topic for commercialization. Fascination with syngas fermentation is trending, as it addresses the limitations of conventional technologies like direct biochemical conversion and Fischer–Tropsch’s method for the utilization of lignocellulosic biomass. A plethora of microbial strains is available for syngas fermentation and PHA production, which could be exploited either in an axenic form or in a mixed culture. These microbes constitute diverse biochemical pathways supported by the activity of hydrogenase and carbon monoxide dehydrogenase (CODH), thus resulting in product diversity. There are always possibilities of enzymatic regulation and/or gene tailoring to enhance the process’s effectiveness. PHA productivity drags the techno-economical perspective of syngas fermentation, and this is further influenced by syngas impurities, gas–liquid mass transfer (GLMT), substrate or product inhibition, downstream processing, etc. Product variation and valorization could improve the economical perspective and positively impact commercial sustainability. Moreover, choices of single-stage or multi-stage fermentation processes upon product specification followed by microbial selection could be perceptively optimized.

## 1. Introduction

The exploitation of fossil deposits for fuels and plastics has been considered a bane by succeeding generations, as these are the concerning causes of the ongoing environmental crisis, and furthermore, it is believed that natural chaos is inevitable if this exploitation continues. Reported possibilities in exploring renewable sources to substitute these fossil-derived products could be a future endeavor. Fermentation technology has opened a window for the production of bioproducts that could be potential alternatives for fossil products. Biofuels and bioplastics from microbial catalysis are biodegradable and eco-friendly alternatives. Cellulosic and lignocellulosic raw materials, which are abundant in municipal and agricultural waste, can be utilized for these bioproducts [1,2,3]. These feedstocks are complex organics that can either undergo biochemical conversion through biocatalysis (mostly microbial catalysis) or be combusted into carbon-rich syngas. The obtained syngas could either be chemically converted to hydrocarbon fuels via a thermochemical process or the syngas could be fermented into valuable bioproducts [2,3,4].

The Fischer–Tropsch (FT) process (thermochemical conversion) and biochemical conversion, the conventional processes for lignocellulosic biomass conversion, are often associated with various limitations (discussed in Section 2). On the other hand, syngas fermentation addresses these limitations of biochemical and thermochemical conversion. Its flexibility towards the H_2_:CO ratio broadens its applicability to a variety of carbonaceous feedstocks and does not require pretreatment for lignocellulosic biomass [2]. As this process exploits the whole cell for catalysis, it is comparatively less sensitive to impurities, toxins, and inhibitors. Moreover, fermentation is accomplished at a mild temperature and pressure. Furthermore, microbial inclusion makes the process more substrate-specific and lessens byproduct formation. The overall operational cost could be minimized through the syngas fermentation process. In a review provided by Daniell et al. (2012), all three processes, i.e., biochemical, thermochemical, and syngas fermentation, were compared and it was concluded that the overall efficiency of syngas fermentation is higher [2]. Biochemical conversion had significant energy loss in converting lignocellulosic biomass to fermentable sugars and energy from lignin could not be captured through this process, whereas both lignin and cellulose could be converted to syngas by gasification. The gasification energy efficiency ranges from 75% to 80%, and it depends on the feedstock composition of carbon, moisture, and ash. Overall plant energy efficiency, which accounts for the energy stored in the feedstock converted to the final product, was 57% in the case of syngas fermentation. However, this overall plant energy efficiency was only 45% for the FT process. These comparative parameters point towards the syngas fermentation process being economically viable for the conversion of lignocellulosic biomass.

**Figure 1:**
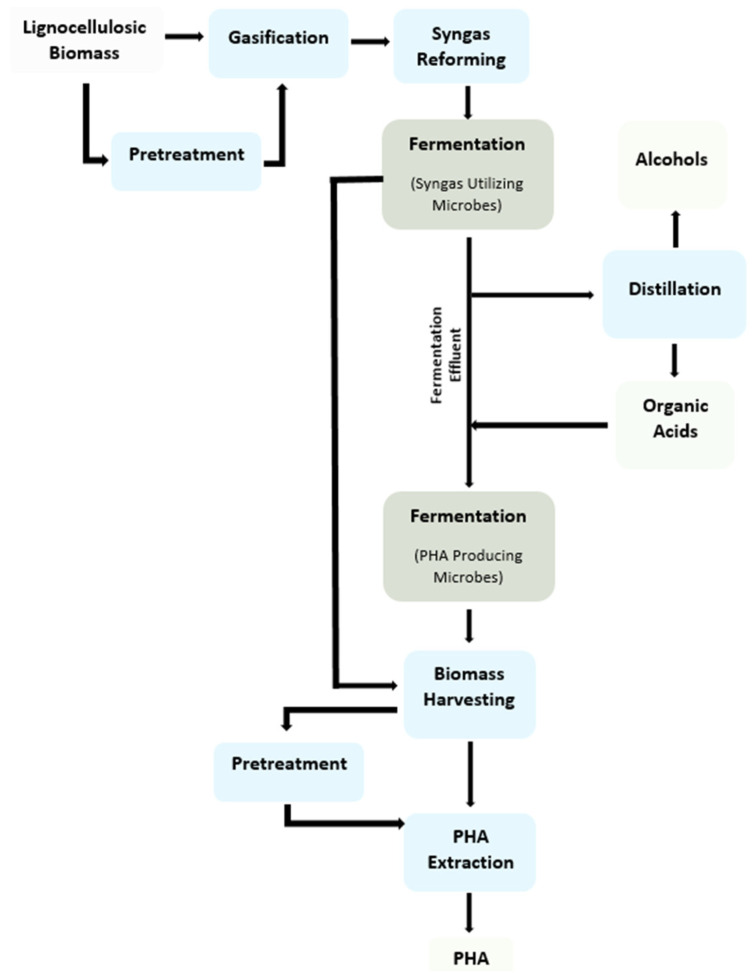
Diagram of the conversion of lignocellulosic feedstock into alcohols, organic acids, and polymers.

Apart from waste utilization, syngas fermentation also provides a perspective for product diversification and valorization. Upon the optimal exploitation of microbes, various products, such as ethanol, higher alcohols, organic acids, and bio-polymers, could result from syngas fermentation [1,2,3,5]. Although most of the acetogenic bacteria can utilize CO and H_2_ to produce alcohols and organic acids, some of them (most from the *Clostridium* species) can produce higher alcohols. Depending on the feed supplement and/or use of chain-elongating bacteria (e.g., *Clostridium kluyveri*) in the culture, the carbon chain in both acid and alcohol could be extended [6,7,8]. Syngas could also be directly fermented to polyhydroxyalkanoates (PHA) through phototrophic bacteria such as *Rhodospirillum rubrum*. Moreover, fermentation effluent from the acetogenic culture could act as a suitable feedstock for polymer production by PHA-producing microbes [9]. Similarly, the type of polymer, i.e., short-chain-length (*scl*) PHA and medium-chain-length (*mcl*) PHA, could be optimized through the carbon chain length of organic acid in the fermentation medium [10]. This paper reviews various aspects of syngas fermentation for the production of fuel and polymers. A scheme for the conversion of lignocellulosic biomass to syngas fermentation products is given in Figure 1.

## 2. Limitations of Conventional Techniques for the Utilization of Lignocellulosic Biomass

The biochemical process for the direct fermentation of lignocellulosic biomass requires undergoing three basic processes that include the release of cellulose from the lignin matrix, the hydrolysis of cellulose into fermentable sugars, and the microbial fermentation of these sugars into bioproducts [2,3,11]. The last two steps of this biochemical conversion could either be performed individually through separate hydrolysis and fermentation (SHF) or achieved in a single process by simultaneous saccharification and fermentation (SSF) [12]. Hydrolysis or saccharification steps involve biocatalysis through cellulase or hemicellulase activity, whereas the whole cell takes part in the fermentation process. The optimal temperature for saccharification with fungal cellulase lies within 50 °C to 55 °C, whereas the optimum is below 35 °C for microbial fermentation [11]. Therefore, SHF fulfills this condition with extra effort and cost for an additional step. On the other hand, SSF could combine both of these steps, but it still has to compromise total yield and productivity, as the process conditions applied could hinder one of the two processes.

Biomass pretreatment mainly aims at setting free the cellulosic and hemicellulosic components from the network of complex lignin and to weaken the crystal structure of the cellulose. Lignin is a tough structural component of plants and some algae, composed of a network of phenylpropane derivatives that covalently binds hemicellulose, therefore entangling cellulose in the matrix. Pretreatment could be achieved through biological, physical, chemical, and physiochemical methods [11]. The biological method involves the degradation of lignin and hemicellulose using microbes, leaving cellulose untouched. Only a few microbes, mainly fungus, are reported to show lignin-degrading enzyme activity [13]. Further, this step is often time-consuming, limiting the rate of the overall process [14]. The physical process involves milling and grinding of the biomass aimed at reducing the particle size and crystallinity of the biomass, thus providing access to the hydrolytic enzyme. This process is associated with high energy costs and thus is considered unsuitable [11]. Chemical pretreatment includes the use of acids and bases, wet oxidation methods, and the use of green solvents. Alkali brings structural modification of lignin by degrading ester and glycosidic linkages [15]. Acids hydrolyze hemicellulose into simple sugars and convert them to furfural derivatives [16]. Wet oxidation decomposes lignin to CO_2_, water, and carboxylic acid. One of the major limitations of these three processes includes post-treatment of the product to neutralize the product, or to remove inhibitors and toxins (by-products of the pretreatment), which may affect the enzymatic and fermentation process ahead [11]. Although green solvents such as ionic liquids and N-methyl morpholine N-oxide (NMMO) can dissolve cellulose with greater efficiency without releasing toxins and inhibitors, they are regarded as costlier alternatives. Like ionic solvents, NMMO has the ability to dissolve a wide range of lignocellulosic biomasses without the need for chemical modification, and due to its low vapor pressure, the maximum solvent could be recovered in the process [11]. Similarly, physiochemical treatments, such as steam explosion, liquid hot water, ammonia fiber explosion, ammonia recycle percolation, and supercritical fluid, have also been reported, but these processes also interfere with one or more limitations of high energy or chemical costs, raw materials, partial degradation of raw material, generation of toxic compounds, and post-treatment process [11].

The Fischer–Tropsch (FT) process is a thermochemical platform that operates at high temperatures and pressure. It relies on the gasification of carbon-rich biomass into syngas, and with the help of metal catalysts such as iron or copper, syngas is thermochemically converted into liquid hydrocarbon fuels. FT has been commercially operated for coal-to-liquid conversion and has been proposed for lignocellulosic biomass conversion. The gasification strategy makes the FT process flexible towards feedstock variety, as a complex biomass including lignin could be accessed [3]. However, several limitations, such as the requirement of a high purity level of syngas to avoid catalyst poisoning; a strict ratio of H_2_:CO gases (2:1 for cobalt-based catalysts) to comply with the stoichiometry requirements of FT reactions, which may be challenging to achieve with feedstock diversity; and the poor selectivity of FT catalysts results in byproducts that require downstream attention, and high temperature and high pressure (over 7 MPa) operation, adding up the capital and operating costs of the process. The purification and composition optimization of syngas accounts for 60% to 70% of the total operational cost in the traditional FT process [2,3].

## 3. Biomass Gasification

Gasification is the process of conversion of carbonaceous biomass into syngas through thermochemical techniques. It is a process where biomass is pyrolyzed in a gasifier using gasifying or oxidizing agents such as pure oxygen, steam, air, or a combination. The final products of this gasification process are termed “syngas,| which contains carbon monoxide (CO), hydrogen (H_2_), and carbon dioxide (CO_2_) as its primary constituents, and depending on the properties of the feedstock, the presence of water (H_2_O), methane (CH_4_), ethane (C_2_H_6_), ethyne (C_2_H_2_), benzene (C_6_H_6_), naphthalene (C_10_H_8_), ammonia (NH_3_), hydrogen cyanide (HCN), nitrogen oxide (NO_x_), sulfur dioxide (SO_2_), hydrogen sulfide (H_2_S), and carbonyl sulfide (COS) have been reported, in order of decreasing concentration [17]. The process may also produce other hydrocarbon contaminants such as tar and ash, the concentrations of which depend on the biomass, gasification system, and process conditions.

To produce syngas, biomass should pass through a three-step process: biomass pretreatment, gasification, and gas cleaning [18]. The pretreatment step is generally associated with the physical conversion of biomass properties to achieve efficient gasification. The processes of drying, pulverization, and pelletization are often conducted to homogenize the biomass and to achieve effective handling and storage efficiency. Moisture in the biomass ranges from 5% to 35%, and a higher moisture content tends to decrease the energy content of syngas. Therefore, drying is required to achieve an optimal moisture concentration of 10% to 15% [18]. Excess moisture is often associated with the incomplete cracking of hydrocarbon in the pyrolysis zone and a temperature reduction in the oxidation zone. Moreover, moisture can decrease the product’s calorific value by decreasing the concentration of CO and increasing the H_2_ and CH_4_ content through a water–gas shift reaction [18]. Mechanical driers like rotatory driers or fluidized bed driers are operated at a temperature above 375 °k to strip moisture from the biomass [18]. Pulverization and pelletization aim at reducing the feed particle size from 20 to 80 mm, thus obtaining homogeneity of the particle. These processes also ease transportation, handling, and storage efforts. It has been reported that the size reduction is associated with the increased efficiency of gasification [2], as it affects the process kinetics and carbon conversion through increased heat and mass transfer.

After pretreatment, gasification can be achieved in key four steps: drying, pyrolysis, oxidation, and reduction [19]. The biomass is dried at 100 °C to 200 °C, after which pyrolysis begins with anaerobic thermal decomposition of the biomass into the vapor of volatile components of carbonaceous feed, leaving char and ash in the residue. During oxidation, char reacts heterogeneously with air introduced in the oxidation zone to produce CO. Oxidation is achieved at a temperature of 975 to 1275 °K and the oxidizing agent includes pure oxygen, air, steam, inert gas (nitrogen and argon), or a mixture. The supply of oxygen in sub-stoichiometric quantities favors the synthesis of CO over CO_2_.

C + O_2_ → CO_2_ (oxidation of carbonaceous fuel)

H_2_ + ½ O_2_ → H_2_O (hydrogen reacting with oxygen in an air blast, producing steam)

Finally, reduction takes place at high temperatures and in the absence of oxygen. Here the feedstock is thermally decomposed, producing char and volatiles. Oxidation reactions are exothermic and the main reactions (Boudouard and steam reactions) in the reduction process require heat to be added. Therefore, the temperature of the gasifier goes down during the reduction reaction. The gasification of char is a rate-limiting step that relies on pressure, the heating rate, the concentration of gasifying species, and biomass properties. Often the Boudouard reaction, which aims to maintain a balance between the reaction of carbon and its gaseous phases (CO and CO_2_), is considered a rate-limiting step of the reduction process [19].

CO_2_ + C →2CO (Boudouard reaction)

C + H_2_O → CO + H_2_ (steam reaction)

CO_2_ + H_2_ → CO + H_2_O (water-shift reaction)

C + 2H_2_ → CH_2_ (methanation)

Gasification units are classified according to the gasifier types (fixed bed, fluidized bed, entrained flow), pressure applied (pressurized or atmospheric), use of oxidizing agents, heating method (direct or indirect), and heating value (low: 4–6 MJ/Nm^3^, medium: 12–18 MJ/Nm^3^, and high: 40 MJ/Nm^3^) [4]. A fixed-bed gasifier is a conventional type that is operated on a small scale. They perform at a temperature of around 1000 °C and can follow updraft or downdraft techniques [2,4]. In an updraft gasifier, the fuel (feedstock) is fed from the top and the gasifying agent from the bottom. Here the temperature is controlled by humidification. The process is initiated by drying the biomass at top of the reactor (drying unit) that operates from 100 °C to 200 °C. Then the fuel is transferred to a pyrolysis chamber, where the biomass is decomposed into volatiles, char, and ash in the absence of oxygen. After, the char falls into the oxidation chamber, where it gets combusted at a temperature of around 1000 °C and the volatiles carried by hot gas are reduced, reaching upwards in the reduction unit. Although this process has higher energy efficiency and produces low particulate matter, the tar concentration in the product may be high, up to 20%, and a portion of tar is carried upward by the product gas. In the downdraft gasifier, raw material is fed from the top and an oxidizing agent is introduced in the oxidation zone, which is above the reduction zone. Here, fuel and product gas flow in the same direction towards the bottom of the reactor. As tar passes through hot charcoal, it is further cracked into product gases. Although there is a significant reduction of tar in this process, energy efficiency is low and particulate content is high [2,4].

Fluidized bed reactors are suitable for large-scale operation and are often used in coal processing. They consist of a bed with finely grained particles, usually silica sand, which is heated along with feedstock and combustion gas. The bed is fluidized by using a gasification agent, which allows uniform mixing and heat distribution among the reactants. The bubbling fluidized bed reactor contains a fine bed just above the grate. The feedstock to be pyrolyzed is placed in the bed and the gasifying agent is blasted through a series of nozzles at the bottom of the bed. The bed temperature is maintained between 700 °C to 900 °C by controlling the air/biomass ratio. The feedstock decomposes into char and volatile gases via contact with a hotbed, and high-molecular-weight compounds like tar are cracked into the product gas. Thus, the process results in low tar concentration in the product gas. In a circulating fluidized bed reactor, the bed material is circulated between the gasifying chamber and the cyclone separator. Separator strips ash from the reaction, whereas char and tar are left behind or are recycled to generate product gas. This type of reactor operates at high temperatures and allows high-throughput operation. They are often used in the decomposition of lignocellulosic waste [2,4].

An entrained flow gasifier is recommended for commercial-scale gasification of coal, especially where product gas needs to undergo FT conversion. Here, fine feedstock particles (dry or slurry) are passed through the top of the reactor along with an oxidizing agent and/or steam. A cloud of steam surrounds the feed and flows concurrently throughout the operation. This process is operated at a high temperature and pressure, generating turbulence in the reactor. The reaction in the entrained flow process occurs at a very high rate, with a carbon conversion efficiency of up to 99.5% [2]. High-molecular-weight compounds are cracked through repeated contact with hot clouds, thus having the ability to produce tar-free product gas. This operation has been considered unsuitable to process lignocellulosic feed, as it requires very fine particles that are less than 0.4 mm in size. However, biomass torrefaction opens a window to the possibility of lignocellulosic feed to be gasified through an entrained flow gasifier [2].

## 4. Syngas Reforming

Gas cleansing is thought to be a major challenge, as it accounts for extra steps and costs. All other components of gas, except CO, CO_2_, and H_2_, are considered impurities, which may affect the fermentation process [1]. Microbial activities are mostly affected by the impurities, as it may lead to cell toxicity, enzyme inhibition, osmotic imbalance, and change in medium pH [1,17,20]. Syngas-fermenting enzymes affected by the impurities (Table 1) include alcohol dehydrogenase (ADH) and amidase being inhibited by NH_3_ (at high concentration); NO (at above 0.004%) inhibiting hydrogenase and affecting ADH [17]; NO_2_ at 1 mol/m^3^ inducing a 5% inhibition in formate dehydrogenase and a 20% inhibition in nitrate reductase [20,21]; thiosulfate sulfurtransferase and L-ascorbate oxidase being affected by an H_2_S concentration > 30 mol/m^3^ and 1 mol/m^3^, respectively [21]; and similarly, carbon monoxide dehydrogenase and ascorbic acid oxidase being affected by carbonyl sulfide and sulfur dioxide, respectively [17]. Tar and nitric oxide have the potential to retard cell growth and enzyme activities during fermentation. On the other hand, H_2_S at a low concentration is considered to be a stimulatory compound, and NO at a concentration above 0.004% could also increase the ethanol-to-acetic-acid ratio, compromising microbial growth [1].

Different approaches that aim to strip impurities from syngas are being reviewed [1]. A cyclone separator is considered a conventional method to remove particulates from syngas. However, the modern approach for hot gas cleaning after gasification involves physical and chemical treatments like cyclones, rotating particle separators, electrostatic filters and scrubbers, and downstream cracking techniques. Some of these reported treatments include tar cracking, which is used for the minimization or removal of high molecular weight hydrocarbons; ammonia removal by water quench scrubbers; sulfur and CO_2_ treatments with amines; zinc oxide/zinc titanates treatment for sulfur removal; and tar minimization by calcined dolomites with a nickel catalytic [23].

## 5. Biochemical Pathways for Syngas Utilization

The introduction of syngas into a suitable fermentation media, inoculated with a selected microbial strain in a controlled condition, initiates syngas fermentation. Carbon-rich gases are converted into an acetyl coenzyme A (acetyl-CoA), a central metabolic intermediate of syngas fermentation, and depending upon the microbial species exploited in the fermentation, this intermediate could be diversified into various products. Along with microbial variation, metabolic pathways and functional enzymes for this conversion differ. Common among these microbes is that they are equipped with the activity of key enzymes, carbon monoxide dehydrogenase (CODH) and hydrogenase (H_2_ase), which enables them to fix syngas [5]. The activity of H_2_ase produces 2 mol of reducing equivalent (H) from 1 mol of H_2_. Similarly, CODH catalyzes the conversion of 1 mol of CO with another mol of H_2_O into a mol of CO_2_ and 2 mol of H through a water–gas shift reaction. Therefore, the produced H is later used to fix carbon molecules from CO and CO_2_ into the biomass and other metabolites. Phototrophic bacteria, acetogenic bacteria, and aerobic carboxydotrops are commonly reported microbes that have shown such ability [5].

**Figure 2:**
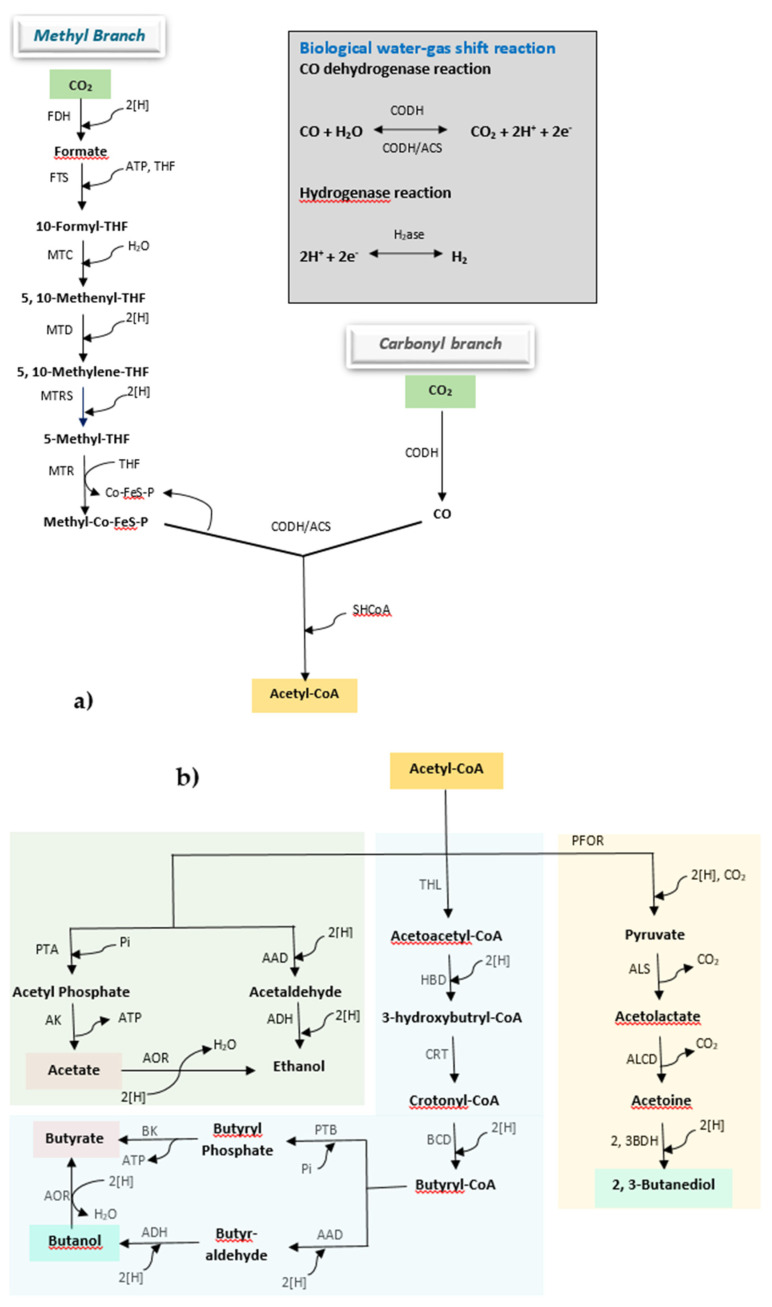
Metabolic processes for syngas conversion to diverse products through the Wood–Ljungdahl pathway (modified from Ciliberti et al. (2020)) [24]. (**a**) Conversion of syngas to acetyl-CoA and (**b**) conversion of acetyl-CoA to fermentation products. Abbreviations: AAD, alcohol/aldehyde dehydrogenase; ACS, acetyl-CoA synthase; ADH, alcohol dehydrogenase; AK, acetate kinase; ALDC, acetolactate decarboxylase; ALS, acetolactate synthase; AOR, aldehyde:ferredoxin-oxidoreductase; BCD, butyryl-CoA dehydrogenase; BK, butyrate kinase; CODH, CO dehydrogenase; Co-Fes-P, corrinoid iron-sulfur protein; CRT, crotonase; FDH, formate dehydrogenase; FTS, formyl-THF synthetase; HBD, 3-hydroxybutyryl-CoA dehydrogenase; HYA, hydrogenase; MTC, methenyl-THF cylcohydrolase; MTD, methylene-THF dehydrogenase; MTR, methyltransferase; MTRS, methylene-THF reductase; PFOR, pyruvate:ferredoxin oxidoreductase; PTA, phosphotransacetylase; PTB, phosphotransbutyrylase; THF, tetrahydrofolate; THL, thiolase.

Following the activity of key enzymes, carbon could be fixed into acetyl-CoA either through the Wood–Ljungdahl or acetyl-CoA pathway (occurs in acetogenic bacteria), or by undergoing the Calvin–Benson–Bassham (CBB) or Calvin cycle (occurs in phototrophic bacteria and carboxydotrophs). Acetogens are a group of anaerobic bacteria in which the Wood–Ljungdahl pathway is active for the conversion of syngas, and they include species such as *Acetobacterium woodii*, *Alkalibaculum bacchi*, *Butyribacterium methylotrophicum*, and many species of the genus *Clostridium*, such as *C. aceticum*, *C. ljungdahlii*, *C. thermoaceticum, C. autoethanogenum*, *C. ragsdalei*, and *C. carboxidivorans* [5]. Based on carbon flow, the Wood–Ljungdahl pathway is divided into a carbonyl stream and a methyl stream (Figure 2a) [2,24,25,26]. In the methyl stream, formate dehydrogenase (FDH) catalyzes the conversion of a mol CO_2_ to a mol of formate utilizing 2 mol of reducing equivalent H. One mol of formate combines with one mol of tetrahydrofolate (THF) to give formyl THF. The formyl THF synthase catalyzes this reaction, where ATP is utilized to fulfill energy for the synthesis. Formyl THF undergoes a series of reactions where it gets reduced by 4 mol of reducing equivalent H to form methyl THF [5]. Corrinoid FeS protein (Co-FeS-P) carries a methyl group from methyl THF to the CODH-ACS complex (ACS: acetyl-CoA synthase), leaving THF free to uptake another formyl group. In the carbonyl stream, either CO_2_ is reduced to a carbonyl group by CODH with expenses of 2 mol of H, or CO is directly converted into a carbonyl group. In the CODH-ACS complex, this carbonyl group combines with the methyl group delivered by Co-FeS-P from the methyl stream and with HSCoA to form acetyl-CoA.

The CBB cycle is operated in phototrophic and carboxydotrophs microbes. Purple non-sulfur bacteria, a syngas fermenting bacterium, is an α-proteobacteria from the *Rhodospirillaceae* family that can grow aerobically in the dark chemoheterotrophically through respiration. During the anaerobic condition, in the presence of light, their intra-cytoplasmic photosynthetic system gets activated, due to which they can switch to a photoheterotrophic or photoautotrophic mode depending upon the availability and unavailability of organic substrates, respectively, as a source of carbon and electrons. *Rhodospirilla rubrum* is reported as being one of the potential species that has the inherent ability to synthesize polyhydroxyalkanoates (PHAs) as a natural polymer by fixing syngas [5]. The reaction initiates with CODH and H_2_ase activity, which generates CO_2_ and H from CO and H_2_O through a water–gas shift reaction and reducing the equivalent H from H_2_, respectively. The CBB cycle plays a vital role in carbon assimilation during the phototrophic mode, whereas in the photoheterotrophic mode this cycle is associated with creating a redox sink balance of reducing equivalents [27]. During the photoheterotrophic mode, other carboxylase enzymes (expect ribulose 1, 5 bisphosphate carboxylase/oxygenase (RUBISCO)) play a vital role in converting inorganic carbon to central percussor molecules.

In the photoautotrophic mode, RUBISCO fixes 3 mol of CO_2_ with 3 mol of ribulose 1, 5 phosphate (R1, 5P) to form 6 mol of 3 phosphoglycerate (3PG), which is then phosphorylated to 1, 3 bisphoshoglycerate (1,3PG) by group transfer from 6 mol of ATP and reduced to 6 mol of glyceraldehyde 3 phosphate (G3P) by 6 mol of NADPH/H^+^. The latter two reactions are catalyzed by 3PG kinase and G3P dehydrogenase, respectively. one mol of G3P exits the cycle and undergoes the glycolysis step to form pyruvate, and then gets converted to acetyl-CoA through decarboxylation, oxidation, and group transfer reactions performed by pyruvate dehydrogenase. On the other hand, the remaining 5 mol of G3P are merged with dihydroxyacetone 3 phosphate to give fructose 1,6 bisphosphate (F1,6P) by aldolase, and this F1,6P is recycled back to R1,5P by a set of reactions catalyzed by transketolase, transaldolase, kinase, epimerase, and isomerase.

Once acetyl-CoA is synthesized, it can be diversified in various products respective to the microbial strains used (Figure 2b) [2]. *M. thermoacetica, A. woodii, C. aceticum*, etc. convert it into acetate through the action of phosphotransacetylase and acetate kinase. Ethanol is produced by the action of alcohol/aldehyde dehydrogenase in *C. autoethanogenum, C. ljungdahlii, C. ragsdalei, A. bacchi*, etc. Similarly, to produce butanol and butyrate, acetyl-CoA is converted to butyryl-CoA by the series of the enzymatic reaction, and it is either converted into butyrate with the action of phosphotransbutyrylase and butyrate kinase, or to butanol by the action of alcohol/aldehyde dehydrogenase, in *C. carboxidivorans, C. drakei, B. methylotrophicum*, etc. On the other hand, acetyl-CoA has to first be converted to pyruvate by the action pyruvate ferredoxin oxidoreductase, and then undergoes synthesis, decarboxylation, and oxidation reactions to produce 2,3-butanediol. Along with ethanol production, *C. autoethanogenum, C. ljungdahlii, C. ragsdalei*, etc. have also shown an ability to produce 2,3-butanediol.

## 6. Process Optimization for Syngas Fermentation

To enlist syngas as a commercial substrate for fermentation, its maximal conversion by the microbial species towards their selective product/s is an utmost requirement. This bioconversion process is limited mostly by the physical processes that include gas diffusivity and its mass transfer in the liquid medium, and by the biochemical processes associated with cell toxicity and the enzyme inhibition led by the concentration of impurities in the syngas. To overcome this hurdle, various approaches have been discussed, which include reactor design and process optimization, medium optimization, syngas reforming, and microbial strain improvements [2,3,23,25,28]. Achievements to date are still behind from a commercial perspective.

Process conditions such as nutrient source and their concentrations, the concentration of gaseous components, flow rates of gas and liquid, medium pH, temperature, pressure, and product concentration play a vital role in microbial growth and product synthesis [2,3]. Nutritional requirements for syngas-fermenting microbes include amino acids, minerals, vitamins, metal co-factors, and reducing agents. As the product profile of the syngas fermentation is found to be higher during the non-growth-promoting stage, the best results could be obtained when these medium components are optimized accordingly for microbial growth and product formation. Limiting nitrogen concentration has shown a significant increase in ethanol concentration [29]. Yeast extract (YE) is one of the most commonly reported medium supplements for syngas fermentation, as it consists of a wide range of organic nutrients, including amino acids, carbohydrates, nucleotides, and carbohydrates [24]. Although it is beneficial for both growth and product synthesis, the higher concentration of YE is often reported to be detrimental for the concentration of products, such as ethanol [30,31,32]. Thi et al. (2020) compared YE, vegetable extract (VE), malt extract (ME), and corn steep liquor (CSL) at 0.5 g/L and 5 g/L concentrations as a supplement in a basal medium for syngas fermentation [32]. It was reported that, at the higher concentration, all the extracts led to a decrease in ethanol production by *C. autoethnogenum*, and this effect was comparatively higher in YE supplementation. On the other hand, acetic acid concentration was increased at the higher concentration of these extracts, except for VE. Liu et al. (2104) also reported increased ethanol productivity from *Alkalibaculum bacchi* when CSL was supplemented in the medium [33]. As YE in the medium is often associated with higher costs for fermentation, ME, VE, and CSL could act as more economical alternatives and could replace standard, more costly, vitamins and mineral components.

Increased substrate consumption and product formation during syngas fermentation have been reported when biochar is introduced in the media [34,35]. Biochar, a carbon-rich material derived from the pyrolysis or gasification of organic matter, contains rich porous structures, functional groups, metals, and minerals. Biochar from switchgrass, forage sorghum, red cedar, and poultry litter was supplemented to the media and compared with a control containing YE only. It was found that char from red cedar and poultry litter increased ethanol production by 16.3% and 58.9%, respectively. Consumption of CO and H_2_ was also increased by 40% and 69%, respectively, compared to media containing YE only, during the fermentation with poultry-litter biochar [34]. Furthermore, it was demonstrated that the use of poultry-litter biochar in syngas fermentation was feasible for enhancing ethanol production in a continuous stirred tank reactor (CSTR) by successfully eliminating costly 4-morpholineethanesulfonic acid (MES) [35]. MES is considered the most expensive constituent, and accounts for more than 90% of the cost of fermentation media. Moreover, Gao et al. (2013) showed statistically that the removal of MES did not affect the growth of *Clostridium ragsdalei* or ethanol production from it at *p* > 0.05 [36].

Ranges of vitamin B present in YE are necessary for the optimal growth and product synthesis of the acetogens [30,37]. Vitamins, although required in lesser quantity, play a vital role in metabolism and its regulation, as they involve prosthetic groups of enzymes. Kundiyana et al. (2011) reported on increased acetic acid and ethanol production by *C. ragsdalei* when vitamin B_12_, calcium pantothenate, and CoCl_2_ were limited in the fermentation medium [37]. Similarly, limiting the mineral nutrient also showed increased product synthesis, but cell growth was compromised [36].

Mineral and trace metals have shown a significant effect as the medium supplement during syngas fermentation. Mineral salts of NH_4_^+^, PO_4_^3−^, S^2−^, and Mg^2+^ were reported to be an essential component of media for *C. ragsdalei*, the absence of which led to a loss of cell mass and product concentration [38]. Similarly, the presence or absence of trace metals that function as co-factors for various metalloenzymes of Wood–Ljungdahl pathways has been reported to interfere with cell mass and productivity [39]. The absence of metal ions of nickel (Ni^2+^), tungstate (WO_4_^-^), cobalt (Co^2+^), iron (Fe^2+^), and molybdate (MoO_4_^−2^) was reported to decrease ethanol production in *C. ragsdalei*. On the other hand, when the concentration of Ni^2+^, zinc (Zn^2+^), selenite (SeO_4_^−^), and WO_4_^−^, were increased from basal level, enhanced alcohol production was observed. Moreover, SeO_4_^−^, WO_4_^−^, and Fe^2+^ were associated with the activity of FDH, and Ni^2+^ and Fe^2+^ were associated with CODH and H_2_ase activity.

A medium pH for syngas fermentation strongly influences microbial metabolism and the end product. With an increasing fermentation period, the medium pH decreases due to acetogenesis, and a decreasing pH favors solventogenesis. In many acetogens, a higher pH (5.0–6.0) is associated with fatty-acid production and cell growth, whereas at a lower pH (4.5–5.0), fatty acids are converted to alcohols [3]. Therefore, a two-stage culture was proposed by Richter et al. (2013), where *C. ljungdhalii* produced 18 g L^−1^ acetate and 5.5 g L^−1^ ethanol during the growth stage at pH 5.5, and ethanol concentration was increased to 20.7 g L^−1^ during the production stage at a pH between 4.5 and 4.8 [40].

Mass transfer of CO and H_2_ from the gas phase to the liquid phase is a critical parameter that determines the availability of these gases for fermentation. Gas–liquid mass transfer (GMLT) is also considered one of the major limitations of syngas fermentation. Approaches taken to address this mass-transfer limitation includes the increment of partial pressure of syngas components, the optimization of reactor design and configurations, the regulation of gas supply systems, the use of electrolytes or a vibrational technique for microbubble generation, an increase in dissolved gas concentration by adsorption through nanoparticles, and suppressed bubble coalescence by using surface-active agents such as nanoparticles, polymers, antifoams, etc. [28].

Process efficiency and the utilization of syngas components are usually associated with the partial pressure of CO in the medium. The higher partial pressure of CO is often reported with increased cell growth and product concentration. According to Henry’s law, gas solubility in the medium increases with increasing partial pressure; thus, mass transfer into the nutrient solution could be enhanced. In a batch process for *C*. *aceticum*, a high CO partial pressure of 204.68 kPa increased the cell concentration and the cell showed good tolerance to CO_2_ [41]. Hurst and Lewis (2010) reported on enhanced ethanol production by *C. carboxidivorans* and a shift in this production from the non-growth stage to the growth stage when the partial pressure of CO was increased from 35.5 to 202.7 kPa [42]. On the other hand, inhibition of enzymes of the metabolic pathway was also reported with increased CO partial pressure. H_2_ase activity of *C. carboxidovorans* was reduced by 97% at a CO partial pressure of 202.7 kPa. An increase in H_2_ase activity of *C. ragsdalei* was reported when H_2_ partial was increased [43]. An enhancement in acetate productivity (7.4 g L^−1^ day^−1^) was observed in *A. woodii* when H_2_ partial pressure was increased up to 170 kPa [44].

Reactor design and configuration have shown substantial effects in GMLT. Syngas fermentation has been studied in different reactor designs, which include a stirred tank reactor, gas/air-lift reactor, loop reactor, trickle bed reactor, membrane reactor, and monolithic biofilm reactor. Munasinghe and Khanal (2010) compared eight reactor configurations for syngas fermentation and reported that the air-lift reactor combined with a 20-micron bulb diffuser resulted in the highest mass transfer coefficient (*k_L_a*) of up to 91.1 h^−1^ [45]. In a comparison performed by Orgill et al. (2013), the highest *k_l_a* (1062 h^−1^) was reported with a hollow fiber membrane reactor (HFMR) [46]. Although *k_L_a* is often used to describe GMLT, it is directly proportional to the flow rate of fluids or the impeller speed, thus resulting in increased power consumption. Therefore, Ungerman and Heindel (2007) described mass transfer performance as the volumetric mass transfer coefficient per unit power input (*k_L_a* P_g_^−1^) [47]. Although several reactor designs have been purposed for syngas fermentation, a continuous stirred tank reactor (CSTR) provides simple and flexible operational control. Therefore, it is commonly used in laboratories and industries. As CSTR could be configured with designs of agitators and aerators, a broad range of mass transfer operations could be performed. In a stirred tank reactor, a dual impeller configuration with a Rushton-type impeller was compared to analyze the mass transfer of CO. Although the dual Rushton-type impeller design presented with the highest *k_L_a* value, the dual design with an axial flow impeller as a top impeller resulted in the highest *k_L_a* P_g_^−1^ value [47]. Furthermore, the addition of activated carbon and nanoparticles of silica to the fermentation medium was also reported to increase the solubility of syngas components and uplift the product concentration [48,49,50].

## 7. Polyhydroxyalkanoates (PHAs) and Their Biochemical Production

PHAs are organic polymers (bio-polyesters) derived from plant or microbial sources and are viewed as a potential alternative to non-biodegradable plastics. They are also considered “green plastics.” They are accumulated in the cytoplasm of an organism during unbalanced growth, especially during nutrition depletion. The major role of PHAs in the microbial cell is to serve as an energy source for an organism during times of starvation. Apart from this, they also exert a protective function for the cells during times of abiotic stresses [51]. PHA granules are spherical in shape and light-refractive, and their core consists of a hydrophobic PHA chain covered with hydrophilic enzymes and structural proteins. PHAs are often reported as macromolecules with helical structures stabilized by the hydrogen bond between the carbonyl group of hydroxyalkanoate monomers [10].

Depending on the number of carbon atoms in the hydroxyalkanoate, PHAs can be either termed as a short-chain length PHAs (*scl*-PHAs) that consist of three to five carbons (two to five when considering glycolate as a PHA building block), or as medium-chain-length PHAs (*mcl*-PHAs) with six or more carbon atoms. The homopolyester of poly(2-hydroxybutyrate) (PHB) is the defined class of *scl*-PHAs, which are highly crystalline and brittle, thus limiting their processing window. Characteristic limitation of PHB materials could be addressed through the synthesis of copolymers and terpolymers by incorporating additional monomers such as 3-hydroxyvalerate (3HV) or 4-hydroxybutyrate (4HB). On the other hand, *mcl*-PHAs are the heteropolyesters of hydroxyalkanoate varieties and express lower crystallinity, higher elasticity, and lower temperature for glass transition in their characteristics. Although homopolyesters of *mcl*-PHAs are reported, they are mostly synthesized by genetically engineered strains [10].

**Figure 3:**
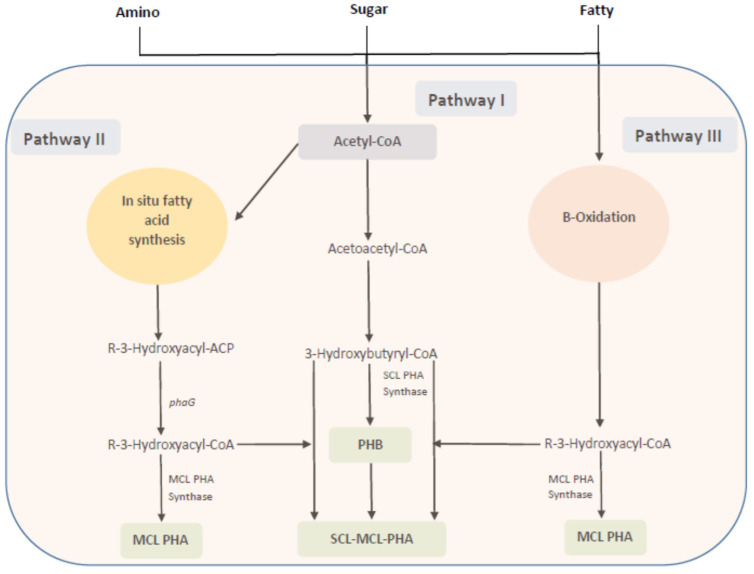
Biochemical pathway for PHA production in a microbial cell Redrawn from [52].

The PHA structures assembled in the microbial cells are dependent on the feed (carbon) source to a greater extent, as those sources are supplied to the relative pathways through which PHA monomers are generated and polymerized by PHA synthase to a final structure. In this regard, carbon sources can be classified into structurally related and unrelated carbon sources. Examples of structurally related carbon sources could be taken as 3HV precursors like propionic acid, valeric acid, levulinic acids, etc., or 4HB precursors like gamma-butyrolactone (structurally related to 3HV and 4HB), 4HB sodium salts, 1,4-butanediole, etc. On the other hand, glucose, lactose, and glycerol, etc., are not structurally similar to PHA; thus, they are taken as unrelated precursors [10,52,53]. Three major PHA synthesis mechanisms in bacteria have been reported (Figure 3), and these mechanisms could be further expanded to 12 different pathways that either have native occurrences or could be tailored in the microbes [52].

Pathway I or the acetyl-CoA to 3-hydroxybutyryl-CoA pathway: Two acetyl-CoAs are merged into acetoacetyl-CoA by the action of β-ketothiolase, which is then successively converted to 3-hydroxybutryryl-CoA by acetoacetyl-CoA reductase. The 3-hydroxybutryryl-CoA is polymerized by *scl*-PHA synthase to PHB. Common precursors for this pathway are sugars, fatty acids, or amino acids, which are converted to acetyl-CoA. *Cupriavidus necator,* which consists of a *scl*-PHA synthase specific for C3-C5 substrates, provides a typical representation for pathway I.

Pathway II or the β-oxidation pathway: This pathway is initialized by the β-oxidation steps where fatty acids are converted to enoyl-CoA, which is then subsequently catalyzed by R-3-hydroxyacyl-CoA hydratase to R-3-hydroxyacyl-CoA. The *mcl*-PHA synthase then polymerizes R-3-hydroxyacyl-CoA to *mcl*-PHA. This pathway is common in the *Pseudomonas* species, such as *P. putida*, *P. olevorans*, and *P. aeruginosa*.

Pathway III or the in-situ fatty acid synthesis pathway: The R-3-hydroxyacyl-ACP (ACP: acyl carrier protein) is an intermediate of fatty-acid synthesis from acetyl-CoA. A key enzyme, 3-hydroxyacyl-ACP transferase, transfers 3-hyroxyacyl from ACP to CoASH to form R-3-hydroxyacyl-CoA, which is then polymerized by *mcl*-PHA synthase to *mcl*-PHA. Precursors could be supplied to *mcl*-PHA synthase by independent operation of the β-oxidation and the in-situ fatty-acid synthesis; therefore, *P. aeruginosa* has the ability to produce random polymers of *mcl*-PHA when grown in structurally non-related substrates.

Although copolymers of *scl*-PHAs, such as PHBV or P3HB4HB, were produced by adding structurally related substrates like propionate, valerate, hexaonate, and 1,4-butanediol to the microbial cultures, these substrates were costly and could also lead to cell toxicity. Therefore, engineering was applied in the metabolic pathways of the cells, which enabled them to produce copolymers of *scl*-PHAs from inexpensive substrates such as glucose. More pathways, in addition to three conventional ones, were accomplished through metabolic engineering. The establishment of a 4HB synthesis pathway via succinic semialdehyde hydrogenase, 4-hydroxybutyrate hydrogenase, 4-hydroxybutyryl-CoA transferase, and PHA synthase is an example of this accomplishment of engineering [52]. Heinrich et al. (2016) engineered *R. rubrum* by introducing genes coding enzymes responsible for producing *mcl*-PHA from *Pseudomonas putida* [54]. He reported that this engineered strain was able to synthesize heteropolymers consisting mainly of 3-hydroxydecanoic acid and 3-hydroxyoctanoic acid (P(3HD-co-3HO)) from artificial syngas.

## 8. Syngas to PHAs

### 8.1. Monoculture

The inherent ability for the conversion of syngas to PHAs is best described in *Rodhospirillum rubrum*. Among the microbes discussed in the above sections, some could inherently ferment syngas but are not able to produce polymers, whereas others have the ability to produce polymers but lack systems of syngas utilization. *R. rubrum* includes CODH and hydrogenase activity to fix syngas, the Calvin cycle to convert syngas to acetyl-CoA, and pathway I (the acetyl-CoA to 3-hydroxybutyryl-CoA pathway) to produce PHA [27]. The *scl*-PHAs are produced by these bacteria, where 3PHB is a dominant monomer of the product. Homopolyester is not the best-described product from *R. rubrum*. During syngas fermentation using *R. rubrum* in RRNCO medium, the product was reported to contain 86% of 3HB and 14% 3HV of total PHA [9]. Similarly, Revelles et al. (2016) reported on obtaining 1% 3HV with the remaining 3HB in RRNCO medium. Even when non-structurally related precursors were used, the presence of 3HV occurrence in the polymer was reported [55]. The PHA compositions were determined in various β-hydroxyalkanoic acids in 550R8AH medium as the sole carbon source during the light-dependent reaction, and the presence of 3HB and 3HV monomers were found from all the carbon sources, irrespective of the amount of carbon in the β-hydroxyalkanoic acids. The ability of *R. rubrum* to generate copolymers also relies greatly on the structurally related precursors. The amount of carbon in the precursor substrate and its concentration greatly influences the amount of carbon in the monomers and the total concentration of those monomers in the PHA chain [55].

The conversion of syngas to PHAs from the conventional technique of monoculture may limit the commercial production process due to the slower growth rate and low conversion efficiency of the associated microbes. From the result of Do et al. (2007), the rate of production of PHAs by *R. rubrum*, supplied with syngas, was approximately 0.0042 g L^−1^ h^−1^ [9]. However, it was reported that PHA content was 38% of cell dry mass (CDM) when syngas from ground corn seed was fed to the culture. Similarly, Koller et al. (2017) conducted a review on various PHA-producing microbes that showed that the rate of production and PHA accumulation by *R. rubrum* was among the lowest [56]. Coming to CO conversion efficiency from the syngas, it was reported to be around 37% and 53% when syngas from microwave-induced pyrolysis and artificial syngas, respectively, were used as the carbon source [27,57]. In that study, the author included acetate in the fermentation medium, which may have interfered with the syngas conversion, as microbial cells have an optimal affinity for acetate as a carbon source.

*Cupriavidus necator* is one of the best-described bacteria for PHA production due to its high productivity. It is a lithotrophic bacteria that can fix atmospheric CO_2_ through the CBB cycle and includes two hydrogenases, soluble and membrane-bound, and the activity of those enzymes generates reducing equivalents for the fixation process [58]. These hydrogenases are tolerant to oxygen, as well as to CO, but they lack CODH activity, which hinders their ability to utilize one of the major components of syngas, i.e., CO. Moreover, the respiratory system of *C. necator* seems to have less sensitivity towards CO, as its cytochromes have a low affinity towards CO [59]. Furthermore, growth inhibition of this species only occurred when the concentration of CO was substantially higher than that of conventional syngas [60]. The genetic engineering approach of introducing CODH-expressing genes in *C. necator* has enabled its ability for conversion of CO to CO_2_, which is utilized for growth [61].

Moreover, strains of *Cupriavidus necator* were illustrated as having a higher efficiency for PHA production of up to 2.84 g L^−1^ h^−1^ when it was fed with glucose and lignocellulosic biomass as the carbon source [62]. Furthermore, this species is reported to accumulate about 80% the dry biomass of PHAs. Supplementation of volatile fatty acids (VFAs) in the fermentation media of *C. necator* has resulted in improved PHA productivity. VFAs such as acetic acid could be produced at an optimal rate from syngas fermenting microbes, and this product of fermentation could act as a substrate for PHA production. On the other hand, acetic acid-producing bacteria such as *Clostridium aceticum* has shown 100% CO conversion efficiency, with an acetic acid productivity of 1.28 g L^−1^ [63]. Considering these results, mixed cultures of two or more efficient species could result in the optimal utilization of syngas with enhanced PHA production. Reports indicate that the optimal concentration of acetic acid of about 0.5 g L^−1^ of fermentation media has led to increased PHA productivity, whereas the increased concentration of this acid also resulted in the inhibition of catalytic pathways [64].

### 8.2. Mixed Culture

A mixed culture using two effective strains, one for syngas conversion and the other for PHA production, inoculated in a single-stage fermentation process, could serve as a sound system. One of the major advantages of this includes process simplification, as it cuts additional units/systems for microbial cultures, microbial contamination issues could be ignored, and high microbial diversity offers increased adaptation capacity, co-fermentation with a mixed substrate, capacity enhancement for continuous processing, etc. On the other hand, this process may also include limitations such as an inhibitory effect resulting from the substrate and/or product toxicity; the optimal process condition for one or another microbe being compromised, thus hindering overall process productivity; an encounter with a dominant species that could suppress the growth of the other; difficulty in downstream processing, etc.

Mixed culture (MC) has been successfully employed in a single-stage process for syngas fermentation (Table 2) and also for PHA production, whereas combinatorial approaches aiming at syngas utilization for PHA production through MC in a single stage are limited. MC exploitation of syngas fermentation has resulted in the generation of higher alcohols. Culturing the strain of ethanol-producing bacteria *Alkalibacterium bacchi* with propionic acid producer *Clostridium propionicum* resulted in the production of ethanol, n-propanol, and n-butanol when syngas was used as a substrate, whereas ethanol was the only outcome during earlier work with a pure culture of *A. bacchi* [6,33]. Other higher alcohols such as octanol [8] and C-6 to C-8 medium-chain carboxylates (MCCAs) are being reported as a result of mixed culture from syngas fermentation [65]. A possible implementation of MCCAs producing bacteria co-culture with PHB producers could result in the production of copolymers (PHA-co-HB) and *mcl*-PHAs, employing the metabolic pathways as discussed in Section 5. MCCA production from syngas is often reported by either culturing *Clostridium kluyveri* in the effluent from the syngas fermentation (two-step process) or by co-culturing syngas-fixing bacteria with *C. kluyveri* (one-step process). *C. kluyveri* is one of the best-described chain-elongating microbes. However, the co-culture of chain-elongating microbes with syngas fermenters has limited efficiency due to the narrow range of pH for co-culture and CO toxicity to the chain-elongating cells [7,8,40,66]. Considering these, two-stage cultures seem to be more efficient. He et al. (2018) enriched MC from biogas sludge to produce MCCAs solely from CO [65]. Enrichment was performed under ecological selection principles. The maximum production rate of *n*-caproate, *n*-heptylate, and *n*-caprylate were 0.276, 0.442, and 0.112 mmol L^−1^ day^−1^, respectively. Furthermore, he concluded that long-term acclimation and high CO partial pressure played a vital role in increasing the tolerance and utilization of CO by chain-elongating microbes such as *Acinetobacter, Alcaligens*, and Rhodobacteraceae.

**Figure 4:**
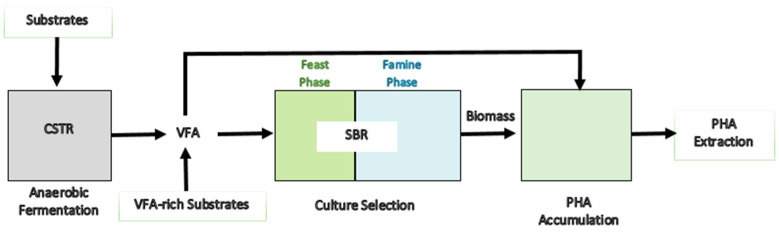
Three-stage process for PHA production through mixed microbial consortia (MMC). Modified from [85]. CSTR: continuous stirred tan reactor, SBR: sequential batch reactor.

Commercial models are successfully being developed with enriched MC for PHA production. Major achievements with MC combine environmental biotechnology methods with industrial biotechnology goals that maneuver an environmental selection approach, where microbes accumulating PHA are enriched by operational conditions imposed on them. Thus, these cultures are termed “mixed microbial consortia” (MMC). Selection and enrichment of microbes with high PHA-synthesizing ability are performed in a sequential batch reactor (SBR) by applying transient conditions. Various microbial enrichment techniques in SBR were reviewed by Kourmentza et al. (2017), and aerobic dynamic feeding (ADF) was stated to be the conventional technique often implied [85]. This process operates on feast/famine cycles, where microbes are initially fed with excess carbon where they accumulate these carbons as PHA and subsequently pass through the famine phase of carbon deficiency. The PHA-accumulating microbes are enriched and selected over others, as they could survive the famine by utilizing the intracellular PHA as a carbon and energy source. Moreover, PHA derived from cellulosic, lignocellulosic, or organic waste through mixed microbial consortia is reported to be the three-stage process (Figure 4) [85]. First is anaerobic fermentation for the production of VFAs, then the effluent from the later process is utilized for the enrichment of PHA-accumulating biomass in SBR, and finally, enriched biomass from the SBR is cultured with the VFA-rich effluent from the anaerobic fermentation for the accumulation of PHA.

For PHA accumulation by the MMC, VFAs are considered the most appropriate precursors, whereas carbohydrates such as glucose are the choice substrate in the fermentation via pure culture. The reason behind this is that MMC tends to accumulate glucose from carbohydrates for glycogen production, thus decreasing PHA concentration [86]. Johnson et al. (2009) reported on acquiring 89% cellular content of PHA, which was estimated at a rate of 1.2 g g^−1^ h^−1^, when acetate (10–30 mM) was supplied in a fed-batch mode [87]. Similarly, Huang et al. (2017) obtained PHA at a rate of 1.21 g L^−1^ d^−1^ in an extended batch culture using synthetic VFAs as a substrate [88]. As inhibition at a high substrate concentration is quite common with VFAs as a substrate during the batch mode, the fed-batch process is often suggested [10,64,85].

The fed-batch mode is further classified into pulse fed and continuous fed. In pulse-fed mode, feed is supplied to the system upon the indication provided by an indicator [10]. Dissolved oxygen and pH are frequently observed as an indicator. Pulse-fed modes are reported to have higher productivity. In a pulse-fed batch process, 33.22 ± 4.2 g of PHA was obtained per kg of organic fraction of municipal solid waste (OFMSW) [89]. Here, OFMSW was initially utilized to produce 151 g kg^−1^ of organic acids by an Anaerobic Percolation Biocell Reactor and PHA comprising a 3HB-to-3HV ratio of 53% to 47% was obtained. Duque et al. 2014 reported obtaining a maximum intracellular PHA (consisting of 3HB and 3HV) content of 56% and 65% from a fermented effluent of cheese whey and sugar-cane molasses, respectively, through the pulse-fed batch approach [90]. Moreover, a continuous feeding process could be chosen for sustained PHA productivity, as it provides appreciable results. High productivities of up to 1.2 g PHA L^−1^ h^−1^ at 72% intracellular PHA content has been reported with a continuous feed system supplement with effluent from molasses fermentation, and this indicates a 2.5–4-fold relative increase in productivities obtained in the pulse-feeding process, with 65% intracellular PHA content [91].

The optimization of multi-stage cultures to carry out syngas fermentation for PHA production shall enhance the productivity and production rate. Here, the first stage is associated with syngas fermentation with high-VFA-yielding acetogens, followed by the culture of PHA producers in the fermentation effluents. The chain-elongating microbes, such as *C. kluyveri,* could be co-cultured in the first stage to produce VFAs with a longer carbon-chain length, thus resulting in the generation of co-polymers and ter-polymers of hydroxyalkanoates in later stages [55,65]. Along with VFA production, syngas fermentation with several acetogens is reported to produce ethanol and higher alcohols, and this account for additional market value (Table 2). Lagoa-Costa et al. (2017) purposed a two-stage fermentation in which the first stage utilized *Clostridium autoethanogenum* to fix syngas (CO:CO_2_:H_2_:N_2_, 30:10:20:40) in an anaerobic condition, where the acetic acid concentration in the final product mixture was 2.66 g/L, along with ethanol and 2,3 butanediol [83]. The second stage was carried out with mixed microbial consortia (MMC) for PHA production in a fed-batch culture, where the product from the first stage was used as a substrate. In this stage, the maximum concentration of PHB obtained was 24% of CDM, where only acetic acid was utilized, leaving ethanol and 2,3-butanediol intact. Later remains of PHA fermentation could be extracted and marketed with distinct commercial value. Similarly, a two-stage process of syngas fermentation to PHA through formate as an intermediate was reported [84]. The first stage of the process included *A. woodii* to fix syngas for formate production by suppressing acetate synthesis through Na^2+^ deprivation. In the second stage, an improved strain of *Methylobacterium extorquens* was used to convert formate to PHA, where 6.5% per CDM of PHB was reported.

## 9. Challenges and Perspective

The production of polymer from syngas fermentation is an emerging technology. Indicators of the maturity of this technology include optimal gas reforming techniques, enhancement of the gas–liquid mass transfer rate, microbial strains with high productivity and tolerance to toxic chemicals from syngas, optimization of the fermentation process, and identification of optimal techniques for the recovery of PHA from the microbial cell. Recent advances in these indicators are discussed in the above sections. Current strategies for PHA production as an alternative to chemically synthesized plastics have encountered substantial cost differences. Moreover, the cost of PHA production by current approaches is three to four times higher than that of conventional plastics [85]. On the other hand, abundant raw material for PHA production from syngas; the demand and use of PHA in the medical sector, food industries, and commercial packaging; and green procurement policies are major forces that could drive commercial PHA production. In a report published in 2021, it was stated that the global PHA market is estimated to reach USD 121 million, from an estimated USD 62 million in 2020, with a compound annual growth rate (CAGR) of 14.2% [92].

An attraction of the syngas fermentation process is that it often delivers multiple products. The production of additional commodities within the existing operational cost could highly influence industrial sustainability. In a techno-economic analysis of syngas-fermenting bio-refinery, producing 50 metric tons of H_2_ gas and 12 metric tons of PHA from the switchgrass, Bents (2007) demonstrated that PHA production from *R. rubrum* was economically viable and technically feasible [74]. The PHA produced from this process of syngas fermentation was less expensive compared to sugar fermentation. Moreover, the revenue generated from the marketed H_2_ gas provided substantial aid to the process operating cost. A similar example reported by Lagoa-Costa et al. (2017), where PHA, ethanol, and higher alcohols were produced through a two-stage culture, is discussed in the above section [83]. Reports on mixed-culture and/or multistage culture for PHA production from syngas are limited, even though this approach has the potential to address multidimensional products.

The availability of numerous microbial strains for syngas fermentation and also for PHA production, as well as variation in their culture condition, drive a combinatorial possibility of these strains. The selection and identification of microbial strains for combination, and their culture optimization, could be a perspective for the mixed-culture mode. Similarly, analyzing the fermentation effluent properties from the first stage of fermentation and studying the effect of those effluents’ components in the second stage of fermentation could be beneficial for multistage fermentation. The incorporation of chain-elongating strains such as *C. kluyveri* could be an additional perspective in mixed culture for syngas fermentation. Successful incorporation would lead to product valorization by producing higher alcohols and co- and ter-polymers of hydroxyalkanoates. The resulting co- and ter-polymers strengthen the characteristics of PHA and occupies a higher end of the market.

## 10. Conclusions

This paper reviewed approaches that are being investigated for the production and fermentation of syngas. Insight has been provided on polymer production from the component of syngas through pure and mixed microbial colonies in single-stage and/or multi-stage cultures. The number of microbial strains associated with syngas fermentation and PHA production along with their major biochemical pathways is being reviewed. Due to the limited choices of microbial strain for direct syngas fermentation to polymers and issues related to their productivity, mixed or multi-stage culture is strongly suggested. Moreover, a focus on product variation and valorization will improve the techno-economical aspect of PHA production through syngas fermentation.

## Figures and Tables

**Table 1:** Effect of syngas impurities on metabolic enzymes.

Impurities	Targeted Enzyme	Effect	References
Ammonia (NH_3_)	Alcohol dehydrogenase (ADH), amidase	ADH inhibition at very high concentrations	[17]
Nitric oxide	Hydrogenase, ADH	100% inhibition of ADH at 0.015 mol%; no effect at 0.004 mol%	[20]
Nitrogen dioxide (NO_2_)	Formate dehydrogenase (FDH), nitrate reductase	5% inhibition for FDH and 20% inhibition of nitrate reductase at 1 mol/m^3^	[21]
Hydrogen sulfide (H_2_S)	Thiosulfate sulfurtransferase (TS), L-ascorbate oxidase (LAO)	>30 mol/m^3^ for TS and 1 mol/m^3^ for 97% inhibition for LAO	[17]
Carbonyl sulfide (COS)	Carbon monoxide dehydrogenase (CODH)		[22]
Sulfur dioxide (SO_2_)	Ascorbic acid oxidase (AAO)		[17]

**Table 2:** Microbial species fermenting the syngas components.

Microorganisms	Growth pH	Growth Temperature (°C)	Substrate	Products	Reference
Monoculture					
*Acetobacterium woodii*	6.8	30	CO/H_2_/CO_2_	Acetate	[45]
*Clostridium ragsdalei*	5.0–7.5	25–40	CO/H_2_/CO_2_	Acetate, ethanol, 2,3-butanediol	[67]
*Clostridium autoethanogenum*	4.5–6.5	20–44	CO/H_2_/CO_2_	Acetate, ethanol, 2,3-butanediol	[67]
*Clostridium ljungdahlii*	4.0–6.0	30–40	CO/H_2_/CO_2_/N_2_	Acetate, ethanol, 2,3-butanediol, formic acid	[40,68]
*Clostridium carboxidivorans*	4.4–7.6	24–42	CO/CO_2_/H_2_	Acetate, ethanol, butyrate, butanol, caproate, hexanol	[69]
*Clostridium drakei*	4.6–7.8	18–42	CO/CO_2_/H_2_	Acetate, ethanol, butyrate, butanol	[69]
*Clostridium scatologenes*	4.6–8.0	18–42	CO/CO_2_/H_2_	Acetate, butyrate	[69]
*Alkalibaculum bacchi*	6.5–10.5	15–40	CO/CO_2_/H_2_	Acetate, ethanol	[33]
*Butyribacterium methylotrophicum*	5.5–6.0	37	CO	Acetate, ethanol, butyrate, butanol	[70]
*Eubacterium limosum*	7.0–7.2	38–39	CO_2_/H_2_	Acetate, butyrate	[71]
*Sporomusa ovate*	5.0–8.1	15–45	CO_2_/H_2_	Acetate, ethanol	[72]
*Peptostreptococcus productus*	7	37	CO/H_2_/CO_2_/N_2_	Acetate	[73]
*Rhodospirillum rubrum*	-	25–30	Syngas	PHA, H2	[57,74]
*Methylocystis Hirsuta*	6.8	25	CH_4_/CO_2_/H_2_S	PHA	[75]
*Synechocystis salina*	6.7	20	CO_2_	PHA	[76]
Thermophiles					
*Acetogenium Kivui*	6.4	66	CO/H_2_/CO_2_/N_2_	Acetate	[77]
*Clostridium thermoaceticum*		55	CO/H_2_/CO_2_/N_2_	Acetate	[77]
*Clostridium thermoautotrophicum*	5.7	36–70	CO_2_/H_2_	Acetate	[78]
*Moorella stamsii*	5.7–8.0	50–70	CO	Acetate, H2	[79]
Mixed culture					
*Alkalibacterium bacchi & Clostridium propionicum*	6.0–8.0	37	CO/CO_2_/H_2_	Acetate, butyrate, propionate, ethanol, butanol, propanol, hexanol	[6]
*Clostridium autoethanogenum & Clostridium kluyveri*	5.5–6.5	37	CO/CO_2_/H_2_	Acetate, butyrate, caproate, ethanol, butanol, hexanol	[7]
*Clostridium lungdahii & Clostridium kluyveri*	5.7–6.4	35	Syngas	Acetate, butyrate, caproate, ethanol, butanol, hexanol, 2,3-butanediol, octanol	[8]
Multi-stage culture					
Elongation of carboxylic acids					
*Clostridium ljungdahlii* (stage 1)	N.A.	N.A.	CO/CO_2_/H_2_	Acetate, ethanol	[80]
Mixed culture (stage 2)	5.5–6.5	30	Fermentation effluent	Acetic acid, butyric acid, caproic acid	
Dicarboxylic (malic) acid production					
*Clostridium ljungdahlii* (stage 1)	5.9	37	CO/H_2_/CO_2_/N_2_	Ethanol, acetate	[81]
*Aspergillus oryzae* (stage 2)	6.5	35	Fermentation effluent	Malic acid	
Lipid production					
*Moorella thermoacetica* (stage 1)	6	60	CO/CO_2_/H_2_	Acetate	[82]
*Yarrowia lipolytica* (stage 2)	7.3	35	Fermentation effluent	Lipids	
Polyhydroxyalkanoate (PHA) production					
*Clostridium autoethanogenum* (stage 1)	5.75	30	CO/H_2_/CO_2_/N_2_	Acetate, ethanol, 2,3-butanediol	[83]
Mixed microbial consortia (MMC) (stage 2)	NA	30	Fermentation effluent	PHA	
PHA production					
*Acetobacterium woodii* (stage 1)	N.A.	N.A.	CO/N_2_	Formate	[84]
*Methylorubrum extroquens* (stage 2)	N.A.	N.A.	Fermentation effluent	PHA	

## Data Availability

Not applicable.
